# The Dietary Index for Gut Microbiota and Live Microbe Intake in Relation to Visceral Fat Obesity: Evidence From NHANES With Vitamin D as a Mediator

**DOI:** 10.1002/fsn3.71530

**Published:** 2026-02-12

**Authors:** Huiling Zheng, Xiaoying He, Jingyi Xiao, Mei Li, Wei Zhang, Yan Wang

**Affiliations:** ^1^ Department of Health Management Centre The First Affiliated Hospital of Sun Yat‐Sen University Guangzhou China; ^2^ Department of Gastroenterology The First Affiliated Hospital of Sun Yat‐Sen University Guangzhou China

**Keywords:** dietary index for gut microbiota, dietary live microbe intake, mediation analysis, NHANES, visceral fat obesity, vitamin D

## Abstract

While the gut microbiota plays a critical role in host metabolism, epidemiological evidence linking microbiota‐targeted dietary patterns to visceral fat obesity (VFO) remains limited. Using data from 8080 adults aged 20–59 years participating in the National Health and Nutrition Examination Survey (NHANES) 2011–2018, we examined the associations of the Dietary Index for Gut Microbiota (DI‐GM) and dietary live microbe intake with visceral fat area (VFA) and VFO, and further explored the potential mediating role of serum vitamin D. Multivariable linear and logistic regression models were applied to assess associations with VFA and VFO, respectively, with restricted cubic spline analyses (RCS) used to evaluate potential non‐linear relationships. Higher DI‐GM quartiles were significantly associated with lower odds of VFO (Q4 vs. Q1, OR = 0.58; 95% CI: 0.43–0.79; *p* < 0.001), independent of demographic, lifestyle, and dietary covariates. Similar inverse associations were observed for higher dietary live microbe intake (G3 vs. G1, OR = 0.66; 95% CI: 0.55–0.81; *p* < 0.001). RCS analyses indicated an approximately linear inverse association between DI‐GM and VFA (nonlinear *p* = 0.097) and a significant nonlinear inverse relationship between MedHi and VFA (nonlinear *p* < 0.001). Mediation analyses revealed that serum vitamin D partially mediated the associations between DI‐GM and VFO (16.8% mediated, *p* < 0.001), and between dietary live microbe intake and VFO (17.0% mediated, *p* < 0.001). In summary, higher adherence to gut microbiota–targeted diets (DI‐GM and live microbe intake) was associated with lower VFA and VFO, with serum vitamin D statistically mediating part of these associations. These findings highlight potential links among diet, vitamin D status, and VFO and warrant further investigation in longitudinal and interventional studies.

## Graphical abstract

Analyzing 8080 U.S. adults, this study found that higher adherence to the Dietary Index for Gut Microbiota and increased dietary live microbe intake were significantly associated with lower visceral fat area and reduced odds of visceral fat obesity. Mediation analysis revealed that serum vitamin D partially mediated these protective associations (approximately 17%). These results highlight the potential of microbiota‐targeted diets and vitamin D status in strategies against visceral adiposity.

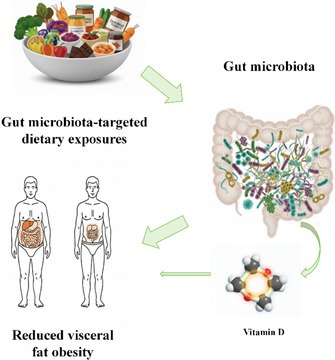

**Abbreviations**
ALT: alanine aminotransferase
BMI: body mass index
DI‐GM: the dietary index for gut microbiota
DM: diabetes mellitus; PA, physical activity
DXA: dual‐energy X‐ray absorptiometry
eGFR: estimated glomerular filtration rate
NCHS: National Center for Health Statistics
NHANES: National Health and Nutrition Examination Survey
OR: odds ratio
PIR: the ratio of family income to poverty
RCS: generalized linear regression models and restricted cubic spline
RCS: restricted cubic spline
SCFAs: short‐chain fatty acids
SFA: subcutaneous fat area
TC: total cholesterol
VDR: vitamin D receptor
VFA: visceral fat area
VFO: visceral fat obesity
WC: waist circumference

## 1 Introduction

VFO, typically defined by excessive VFA, has become a pressing global health concern (Kim et al. 2022). Unlike general obesity measured by body mass index (BMI), VFO is more strongly associated with cardiometabolic disorders, including insulin resistance, type 2 diabetes, and cardiovascular disease (Sandeep et al. 2007; Matsuzawa et al. 1995; He et al. 2010). Visceral adipose tissue is metabolically active and secretes a range of pro‐inflammatory cytokines and adipokines that contribute to systemic inflammation, endothelial dysfunction, and insulin resistance, thereby playing a pivotal role in the pathogenesis of metabolic and cardiovascular diseases (Tanaka et al. 2018; Kolb 2022; Item and Konrad 2012). Epidemiological data suggest that the prevalence of VFO is rising worldwide, including in individuals with a normal BMI—a phenomenon often referred to as “normal‐weight obesity” (Zheng et al. 2023). Given its insidious nature and strong links to adverse health outcomes, identifying modifiable dietary and lifestyle factors associated with VFO is of paramount importance for public health and clinical prevention strategies.

Growing evidence suggests that the gut microbiota plays a pivotal role in energy metabolism, immune regulation, and adipose tissue distribution (Turnbaugh et al. 2006; Ridaura et al. 2013; Tilg and Moschen 2014). Alterations in gut microbial composition—termed dysbiosis—have been closely linked to obesity‐related phenotypes, including visceral fat accumulation (Dao et al. 2016; Bouter et al. 2017). Diet is a major modifiable factor shaping the gut microbiota, with certain dietary patterns known to promote a healthier microbial profile that may protect against obesity and metabolic dysfunction (Conlon and Bird 2014; Sonnenburg and Sonnenburg 2014). Recently, two dietary indicators have emerged to quantify microbiota‐targeted nutritional exposures: DI‐GM, which reflects the intake of microbiota‐accessible carbohydrates and polyphenols, and dietary live microbe intake, derived from consumption of traditionally fermented or raw foods containing viable microorganisms (Kase et al. 2024; Hill et al. 2023; Marco et al. 2022). Growing evidence from both our previous research (Liu et al. 2025) and other observational studies (Hill et al. 2023; Chen et al. 2024) has demonstrated that dietary live microbe intake is associated with BMI and general obesity status; existing studies have primarily focused on these single microbial exposures rather than broader gut‐microbiota–related dietary patterns. In particular, the potential role of indices such as DI‐GM in relation to VFO, a metabolically harmful obesity phenotype, remains largely unexplored.

While dietary patterns influence obesity through gut microbiota modulation, emerging evidence suggests that micronutrients may also play a crucial mediating role in this relationship. Vitamin D, a fat‐soluble secosteroid with both endocrine and immunomodulatory functions, has emerged as a potential link between diet, gut microbiota, and obesity (Luthold et al. 2017; Guo et al. 2022; Boughanem et al. 2023). Vitamin D status is influenced not only by sunlight exposure and dietary intake but also by intestinal absorption and microbial metabolism (Jones et al. 2013). Deficiency in vitamin D has been associated with increased visceral fat accumulation, systemic inflammation, and insulin resistance (Autier et al. 2014; Holick 2007). Additionally, recent studies suggest that certain gut microbial compositions may modulate vitamin D metabolism, indicating a complex bidirectional relationship (Boughanem et al. 2023). These findings point to a possible mediating role of vitamin D in the diet–microbiota–obesity axis.

Despite these insights, few population‐based studies have explored whether vitamin D mediates the relationship between microbiota‐targeted dietary patterns and visceral fat obesity. To address these gaps, we utilized data from NHANES to (1) evaluate the associations between DI‐GM and dietary live microbe intake with VFO, and (2) assess the potential mediating role of serum vitamin D levels in these associations. We hypothesized that (1) higher DI‐GM scores (≥ 7 points) and greater dietary live microbe intake (≥ median) would be associated with 30%–50% lower odds of VFO compared to the lowest categories, and (2) serum vitamin D would mediate 10%–20% of these protective associations.

## 2 Methods

### 2.1 Study Population

**FIGURE 1:**
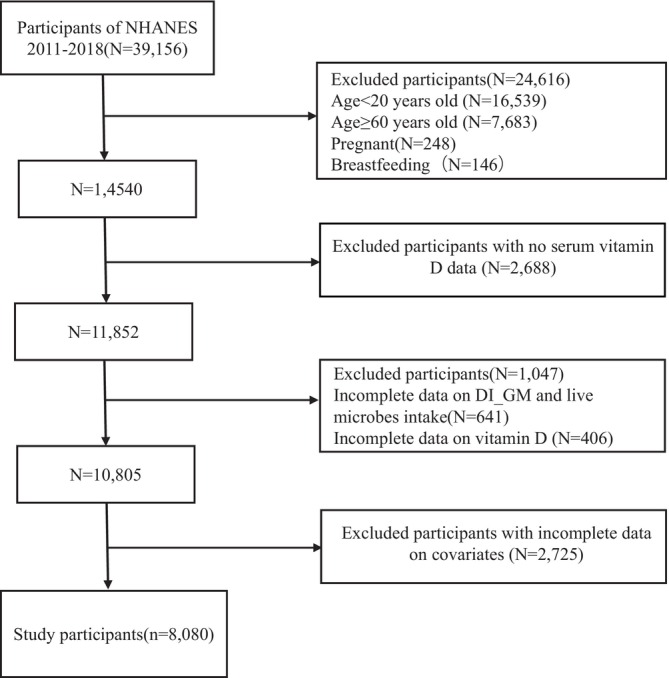
Participant flowchart.

This study utilized data from adult participants aged 20 to 59 years enrolled in four consecutive NHANES cycles spanning 2011 to 2018, as dual‐energy X‐ray absorptiometry (DXA) measurements were only available for individuals under 60 years of age (Figure 1). We excluded individuals who were pregnant or breastfeeding during the survey period, as well as those with missing data on key variables, including VFA, DI‐GM, dietary intake of live microbes, and serum vitamin D. Participants with incomplete information on relevant covariates were also omitted from the final analytic sample. NHANES protocols were approved by the Research Ethics Review Board of the National Center for Health Statistics (NCHS), and all participants provided written informed consent. As this investigation involved secondary analysis of publicly available, de‐identified data, additional institutional review board approval was not required.

Based on prior literature indicating a VFO prevalence of approximately 38% (Kim et al. 2022; Chaudhary and Sharma 2023) and expecting an odds ratio of 0.6 for the highest versus lowest dietary categories, our sample size of 8080 participants provided > 90% power to detect significant associations at *α* = 0.05.

### 2.2 Assessment of Visceral Fat Obesity

VFA and subcutaneous fat area (SFA) were measured using DXA, which provides detailed quantification of soft tissue composition. The visceral‐to‐subcutaneous fat ratio (VSR) was calculated by dividing VFA by SFA. VFO was defined using sex‐specific cutoff based on established criteria: in men, VFO was identified as VFA ≥ 130 cm^2^ or VSR ≥ 1.0; in women, as VFA ≥ 85 cm^2^ or VSR ≥ 0.5 (Kim et al. 2022).

### 2.3 Assessment of DI‐GM

The DI‐GM was calculated based on the scoring system proposed by Kase et al. (2024), using data from the 24‐h dietary recall and incorporating 14 food groups or nutrients. Each dietary component was classified as either favorable or unfavorable according to its reported association with gut microbial diversity. For favorable components, participants received 1 point if their intake met or exceeded the sex‐specific median; otherwise, they received 0 points. For unfavorable components, 1 point was assigned if intake was below the sex‐specific median, or, in the case of fat intake, if less than 40% of total energy came from fat; otherwise, 0 points were given. The total DI‐GM score ranged from 0 to 13, with higher scores indicating a more favorable dietary pattern for gut microbial health. Participants were categorized into quartiles according to the empirical distribution of total scores: Q1 (0–3), Q2 (4), Q3 (5–6), and Q4 (≥ 7), with the lowest quartile serving as the reference category (Shu et al. 2025). These quartile ranges reflect the underlying population distribution of a discrete dietary index rather than arbitrarily defined cut‐offs.

### 2.4 Dietary Intakes of Live Microbe Category

NCHS collaborated with the US Department of Agriculture (USDA) to compare the 24‐h dietary data against the USDA Food and Nutrient Database, estimating nutrient and energy intakes. Sanders devised a method to quantify the live microbial content (per gram) in 9388 food codes across 48 subgroups within the NHANES database (Ridaura et al. 2013). Four experts in the field (MLM, MES, RH, and CH) categorized the live microbial content of foods into three levels: low (Lo; < 10^4^ CFU/g), medium (Med; 10^4^–10^7^ CFU/g), and high (Hi; > 10^7^ CFU/g). Any disagreements among the experts were resolved through internal discussions and external consultations with Fred Breidt, a microbiologist at the USDA Agricultural Research Service.

To capture a broader spectrum of habitual dietary exposure to live microbes in population‐based analyses, a composite MedHi category was constructed, comprising foods classified as medium or high in live microbial content. This approach has been adopted in prior epidemiological studies (Huo et al. 2024; Zhang et al. 2025) and is intended to reflect overall dietary patterns rather than precise quantification of viable microbes at the point of consumption.

In this study, individuals were classified into three groups based on their MedHi consumption levels to assess live microbe ingestion: G1 (no MedHi food intake), G2 (MedHi foods consumed above zero but below the median level), and G3 (MedHi foods consumed at or above the median level).

### 2.5 Vitamin D Assessment

Serum vitamin D concentrations were measured at the NHANES central laboratory using standardized protocols to minimize assay variability. From 2007 to 2018, quantification was performed via ultra‐high performance liquid chromatography–tandem mass spectrometry (UHPLC–MS/MS), ensuring consistency across survey cycles. Serum vitamin D levels were treated both as a continuous variable and a categorical variable. Consistent with established clinical guidelines, vitamin D deficiency was defined as serum 25‐hydroxyvitamin D < 50 nmol/L (20 ng/mL) (Holick et al. 2011).

### 2.6 Covariates

Potential confounders were evaluated as covariates. Questionnaires collected information on age, sex, waist circumference (WC), Waist‐to‐height ratio, race (mexican american, non‐hispanic black, non‐hispanic white, other hispanic, other race), educational level (college or above, high school or GED, less than high school), family income‐to‐poverty ratio (PIR) (< 1.3, 1.3–3.5, > 3.5), smoking status (never, former, current smoker), and physical activity (PA) (< 500MET‐min/week, ≥ 500MET‐min/week). Drinking status was categorized into four groups: never (fewer than 12 drinks in a lifetime), former (no drinking in the past year but ≥ 12 drinks previously), light (up to 1 drink per day for women or 2 drinks per day for men in the past year), and heavy (more than 1 drink per day for women or 2 drinks per day for men on average over the past year). Laboratory analysis covariates included serum total cholesterol (TC), alanine aminotransferase (ALT), and estimated glomerular filtration rate (eGFR). Individuals were classified as hypertensive based on one or more of the following criteria: a self‐reported doctor's diagnosis of hypertension, current use of antihypertensive medications, a systolic blood pressure of 140 mmHg or higher, or a diastolic blood pressure of 90 mmHg or higher. Diabetes was defined based on any of the following criteria: a 2‐h plasma glucose level ≥ 11.1 mmol/L, an HbA1c level ≥ 6.5%, a fasting plasma glucose (FPG) level ≥ 7.0 mmol/L, current use of diabetic medications or insulin, or a self‐reported physician diagnosis. Daily dietary variables assessed included dietary vitamin D intake, total energy, and protein.

## 3 Statistical Analysis

All analyses incorporated NHANES sampling weights, strata, and primary sampling units to account for the complex, multistage survey design and ensure national representativeness, following NHANES analytic guidelines. Baseline characteristics between participants with and without VFO were compared using Rao–Scott *χ* ^2^ tests for categorical variables and survey‐weighted linear regression for continuous variables.

Multivariable logistic regression was applied to estimate the odds of VFO in relation to quartiles of DI‐GM and three‐level categories of dietary live microbe intake, defined according to MedHi food consumption: G1 (no intake), G2 (intake > 0 but < median), and G3 (intake ≥ median). Linear regression models were used to examine associations between these dietary exposures and VFA as a continuous variable. The lowest category in each exposure was used as the reference. Results were presented as odds ratios (ORs) or β coefficients with 95% confidence intervals (CIs).

Four hierarchical models were fitted to progressively adjust for potential confounders: Model 1: unadjusted; Model 2: adjusted for age and sex; Model 3: further adjusted for race, PIR, educational attainment, smoking status, alcohol use, physical activity (PA), estimated glomerular filtration rate (eGFR), alanine aminotransferase (ALT), total cholesterol (TC), total energy intake, protein intake, vitamin D intake, hypertension, and diabetes mellitus (DM); Model 4: additionally adjusted for serum vitamin D. These covariates were selected based on their established associations with both dietary patterns and visceral adiposity in previous literature, following directed acyclic graph analysis to minimize confounding while avoiding overadjustment.

In addition, mediation analyses were conducted to assess the potential mediating role of vitamin D in the association between dietary exposures and visceral fat accumulation. All statistical analyses were conducted using R (version 4.4.3). A two‐sided *p* < 0.05 was considered statistically significant.

## 4 Results

### 4.1 Participant Characteristics

A total of 8080 adults aged 20–59 years were included in the final analysis (Table 1). The weighted mean age of the study population was 39.1 years (range: 20–59 years), and 45.2% were female. Participants with VFO were significantly older (44.4 vs. 35.7 years, *p* < 0.0001), more likely to be female (56.1% vs. 38.1%), and had higher mean BMI values (33.1 vs. 25.8 kg/m^2^) compared to those without VFO (*p* < 0.0001). In addition, participants with VFO had markedly greater WC (108.99 vs. 90.05 cm, *p* < 0.0001) and higher waist‐to‐height ratios (0.65 vs. 0.53, *p* < 0.0001). Additionally, individuals with VFO exhibited higher TC, ALT levels, and lower eGFR, all with statistically significant differences.

**TABLE 1:** Baseline characteristics of participants stratified by visceral fat obesity status.

Variables	Visceral fat obesity			*p*
	Total	No	Yes	
Age	39.12 (0.30)	35.67 (0.36)	44.42 (0.32)	< 0.0001
Sex				
Female	3686 (45.20)	1818 (38.11)	1868 (56.12)	< 0.0001
Male	4394 (54.80)	3152 (61.89)	1242 (43.88)	
BMI	28.68 (0.14)	25.82 (0.12)	33.06 (0.21)	< 0.0001
WC (cm)	97.48 (0.32)	90.05 (0.30)	108.99 (0.39)	< 0.0001
Waist‐to‐height ratio	0.57 (0.00)	0.53 (0.00)	0.65 (0.00)	< 0.0001
TC (mmol/L)	4.97 (0.02)	4.80 (0.02)	5.23 (0.04)	< 0.0001
Alt (U/L)	26.34 (0.31)	24.25 (0.37)	29.55 (0.64)	< 0.0001
eGFR (mL/min/1.73m2)	101.14 (0.42)	102.85 (0.52)	98.50 (0.46)	< 0.0001
Protein intake (g/L)	88.81 (0.74)	92.53 (1.03)	83.10 (1.18)	< 0.0001
Energy intake (kcal/d)	2290.36 (13.96)	2373.10 (18.88)	2162.98 (27.37)	< 0.0001
Vitamin D intake (mcg/d)	4.61 (0.10)	4.86 (0.14)	4.24 (0.13)	0.002
Race				
Mexican American	1113 (9.73)	506 (8.18)	607 (12.13)	< 0.0001
Non‐Hispanic Black	1746 (10.95)	1191 (12.23)	555 (8.97)	
Non‐Hispanic White	3061 (63.11)	1804 (61.88)	1257 (64.99)	
Other Hispanic	770 (6.63)	458 (6.74)	312 (6.46)	
Other Race	1390 (9.58)	1011 (10.97)	379 (7.45)	
Education				
College or above	5085 (67.93)	3253 (70.14)	1832 (64.53)	< 0.0001
High school or GED	2632 (29.42)	1556 (27.89)	1076 (31.77)	
Less than high school	363 (2.65)	161 (1.97)	202 (3.70)	
PIR				
< 1.35	2390 (21.82)	1390 (23.02)	1000 (23.40)	0.97
1.35–3.5	2581 (30.76)	1588 (32.69)	993 (32.62)	
> 3.5	2512 (41.59)	1622 (44.29)	890 (43.99)	
Alcohol consumption				
Never	928 (8.77)	533 (8.30)	395 (9.50)	0.002
Former	684 (7.50)	337 (6.36)	347 (9.25)	
Light	4282 (54.79)	2711 (55.33)	1571 (53.96)	
Heavy	2186 (28.94)	1389 (30.01)	797 (27.29)	
Smoking status				
Never	4850 (58.60)	3041 (60.93)	1809 (55.01)	< 0.001
Former	1406 (20.36)	787 (18.29)	619 (23.54)	
Now	1824 (21.04)	1142 (20.77)	682 (21.45)	
Vitamin D deficiency				
No	5257 (74.53)	3273 (76.69)	1984 (71.19)	< 0.0001
Yes	2823 (25.47)	1697 (23.31)	1126 (28.81)	
DI‐GM				
Q1	1976 (22.23)	1192 (21.82)	784 (22.87)	< 0.001
Q2	2085 (24.34)	1239 (22.70)	846 (26.86)	
Q3	3073 (40.16)	1915 (40.65)	1158 (39.41)	
Q4	946 (13.27)	624 (14.83)	322 (10.86)	
MedHi group				
G1	3042 (33.96)	1823 (31.96)	1219 (37.03)	< 0.001
G2	911 (11.07)	537 (10.64)	374 (11.72)	
G3	4127 (54.98)	2610 (57.40)	1517 (51.26)	
PA				
METminweek	6969 (86.97)	4442 (90.61)	2527 (81.38)	< 0.0001
≥ 500MET‐min/week	1111 (13.03)	528 (9.39)	583 (18.62)	
Hypertension				
No	5820 (73.49)	3978 (82.03)	1842 (60.34)	< 0.0001
Yes	2260 (26.51)	992 (17.97)	1268 (39.66)	
DM				
No	7262 (92.18)	4750 (96.73)	2512 (85.16)	< 0.0001
Yes	818 (7.82)	220 (3.27)	598 (14.84)	

Abbreviations: ALT: alanine aminotransferase; DI‐GM: Dietary Index for Gut Microbiota; DM: diabetes mellitus; eGFR: estimated glomerular filtration rate; PA: physical activity; PIR: poverty income ratio; TC: total cholesterol; WC: waist circumference.

Dietary and biochemical profiles also differed between groups. Participants with VFO had lower protein intake (83.10 vs. 92.53 g/day), reduced total energy intake (2162.98 vs. 2373.10 kcal/day), and lower vitamin D intake (4.24 vs. 4.86 mcg/day), with all comparisons reaching statistical significance.

### 4.2 Association Between DI‐GM, Dietary Live Microbe Intake, and VFO

Multivariable logistic regression analysis revealed significant inverse associations between both DI‐GM and dietary live microbe intake and VFO (Table 2).

**TABLE 2:** Association between DI‐GM, MedHi and VFO.

	Model 1 OR (95% CI)	*p*	Model 2 OR (95% CI)	*p*	Model 3 OR (95% CI)	*p*	Model 4 OR (95% CI)	*p*
DI_GM groups								
Q1	1.00 (Reference)		1.00 (Reference)		1.00 (Reference)		1.00 (Reference)	
Q2	1.13 (0.96, 1.33)	0.14	1.08 (0.91, 1.29)	0.36	1.05 (0.83, 1.33)	0.68	1.07 (0.85, 1.36)	0.53
Q3	0.92 (0.81, 1.06)	0.26	0.74 (0.65, 0.85)	**< 0.0001**	0.76 (0.63, 0.91)	**0.004**	0.79 (0.66, 0.96)	**0.02**
Q4	0.70 (0.56, 0.87)	**0.002**	0.44 (0.34, 0.56)	**< 0.0001**	0.51 (0.38, 0.68)	**< 0.0001**	0.58 (0.43, 0.79)	**< 0.001**
*p* for trend		0.09		**< 0.0001**		**0.006**		**0.04**
MedHi groups								
G1	1.00 (Reference)		1.00 (Reference)		1.00 (Reference)		1.00 (Reference)	
G2	0.95 (0.77, 1.17)	0.63	0.92 (0.73, 1.15)	0.45	0.87 (0.67, 1.13)	0.87	0.88 (0.67, 1.14)	0.32
G3	0.77 (0.67, 0.88)	**< 0.001**	0.59 (0.51, 0.68)	**< 0.0001**	0.62 (0.51, 0.75)	**< 0.0001**	0.66 (0.55, 0.81)	**< 0.001**
*p* for trend		**0.044**		**< 0.001**		**0.008**		**0.04**

*Note:* Model 1 was crude model. Model 2 was adjusted for age and gender. Model 3 was adjusted for Model 2, and race, PIR, educational level, alcohol use, smoking status, eGFR, ALT, TC, protein intake, energy intake, vitamin D intake, hypertension, DM, and PA. Model 4 was adjusted for Model 3, and serum vitamin D.

Abbreviations: ALT: alanine aminotransferase; CI: confidence interval; DI‐GM: Dietary Index for Gut Microbiota; DM: diabetes mellitus; eGFR: estimated glomerular filtration rate; OR: odds ratio; PA: physical activity; PIR: poverty income ratio; TC: total cholesterol. Values in bold indicate statistical significance (*p* < 0.05).

Participants in the highest quartile of DI‐GM (Q4) had significantly lower odds of VFO compared to those in the lowest quartile (Q1) across all models. After adjusting for demographic and lifestyle factors (Model 3), the odds ratio (OR) for Q4 was 0.51 (95% CI: 0.38–0.68; *p* < 0.0001), and this association remained robust after additional adjustment for serum vitamin D (Model 4: OR = 0.58, 95% CI: 0.43–0.79; *p* < 0.001). A significant linear trend was observed across increasing DI‐GM quartiles (*P* for trend = 0.04 in Model 4), indicating a dose–response relationship.

Similarly, higher intake of live microbes was inversely associated with VFO. Compared to participants without any MedHi food intake (G1), those in the highest consumption group (G3) exhibited significantly reduced odds of VFO (Model 3: OR = 0.62, 95% CI: 0.51–0.75; *p* < 0.0001; Model 4: OR = 0.66, 95% CI: 0.55–0.81; *p* < 0.001). A significant trend across categories was also noted (*p* for trend = 0.04 in Model 4).

### 4.3 Association of DI‐GM and MedHi Intake With VFA

Linear regression analyses revealed significant inverse associations of both the dietary index for DI‐GM and dietary live microbe intake (MedHi) with VFA (Table 3). In the fully adjusted model (Model 4), higher DI‐GM scores were associated with lower VFA (β = −2.58, 95% CI: −3.57 to −1.59, *p* < 0.0001). Similarly, each 100g increase in MedHi intake was significantly associated with a 2.17 cm^2^ reduction in VFA (95% CI: −3.08 to −1.26, *p* < 0.0001).

**TABLE 3:** Regression analysis showing the association between DI‐GM, MedHi (per 100g) with VFA.

Variables	Model 1	*p*	Model 2	*p*	Model 3	*p*	Model 4	*p*
DI‐GM	−2.66 (−3.82, −1.50)	**< 0.0001**	−3.94 (−4.92, −2.96)	**< 0.0001**	−3.15 (−4.08, −2.22)	**< 0.0001**	−2.58 (−3.57, −1.59)	**< 0.0001**
MedHi (per 100g)	−2.77 (−3.74, −1.81)	**< 0.0001**	−3.42 (−4.32, −2.52)	**< 0.0001**	−2.61 (−3.62, −1.60)	**< 0.0001**	−2.17 (−3.08, −1.26)	**< 0.0001**

*Note:* Models 1–4 were adjusted as described in Table 2. Values in bold indicate statistical significance (*p* < 0.05).

**FIGURE 2:**
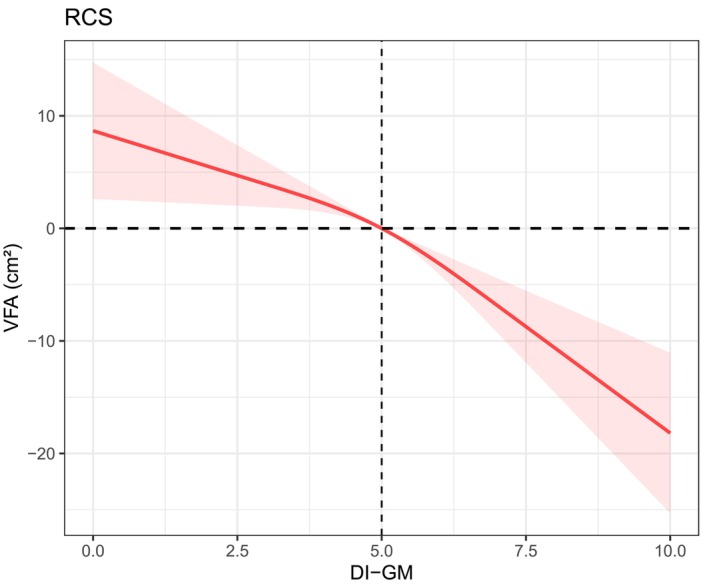
RCS plot of the association between DI‐GM and VFA.

**FIGURE 3:**
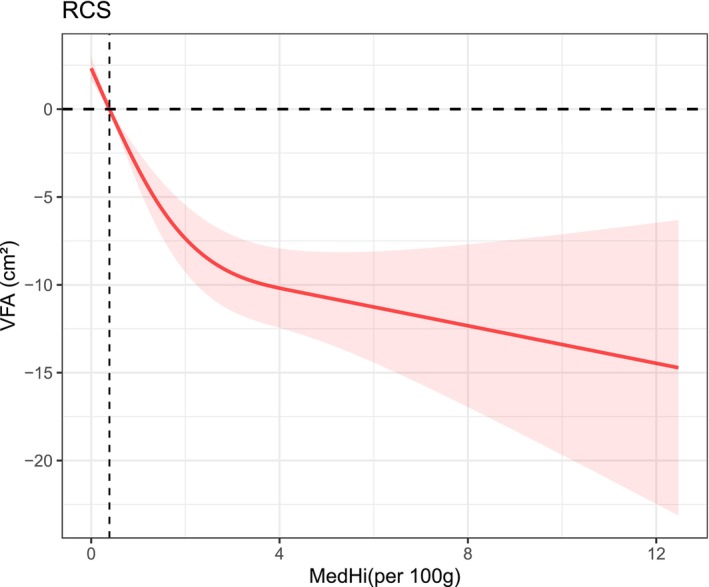
RCS plot of the association between MedHi intake (per 100 g) and VFA.

To further explore potential nonlinear relationships, restricted cubic spline (RCS) models were applied. For DI‐GM, the overall association with VFA was statistically significant (*p overall* < 0.001) (Figure 2), while the test for nonlinearity did not reach statistical significance (*p* nonlinear = 0.0967) (Figure 3), suggesting a predominantly linear inverse relationship. In contrast, MedHi intake demonstrated both significant overall association (*p overall* < 0.001) and significant nonlinearity (*p* nonlinear < 0.001), indicating a nonlinear inverse relationship with VFA.

### 4.4 Mediation Analysis

To investigate whether serum vitamin D levels mediated the association between gut microbiota–targeted diet and VFO, mediation analysis was performed using survey‐weighted models.

**FIGURE 4:**
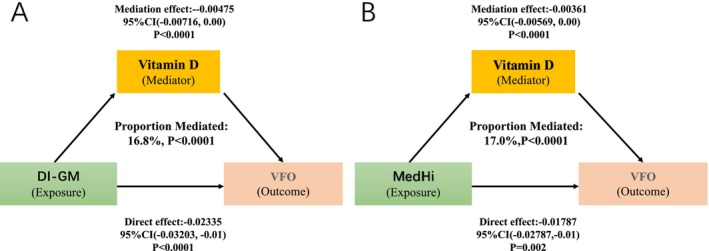
Mediation analysis models for DI‐GM (A) and live microbe intake (B) with vitamin D as a mediator of VFO.

For the DI‐GM score, vitamin D significantly mediated its association with VFO (Figure 4A). The average mediation effect was −0.00475 (95% CI: −0.00716 to 0.000, *p* < 0.001), and the average direct effect was −0.02335 (95% CI: −0.03203 to −0.01467, *p* < 0.001). The total effect was −0.02810 (95% CI: −0.03665 to −0.01955, *p* < 0.001), with 16.78% (95% CI: 9.8% to 28%) of the effect mediated through vitamin D.

Similarly, for dietary live microbe intake (MedHi), vitamin D significantly mediated its inverse association with VFO (Figure 4B). The average mediation effect was −0.00361 (95% CI: −0.00569 to 0.000, *p* < 0.001), and the average direct effect was −0.01787 (95% CI: −0.02787 to −0.00787, *p* = 0.002). The total effect was −0.02148 (95% CI: −0.03150 to −0.01145, *p* < 0.001), with 17.0% (95% CI: 7.6% to 34%) of the effect mediated through vitamin D.

These results indicate that vitamin D status partially mediates the protective associations of both DI‐GM and MedHi intake with visceral fat obesity.

### 4.5 Sensitivity Analyses

To assess the robustness of our primary findings, we conducted a series of sensitivity analyses. First, among participants with BMI < 30, both DI‐GM and MedHi intake remained inversely associated with visceral fat obesity, as shown in Table S1. Similarly, in the analysis of general obesity (BMI ≥ 30), higher DI‐GM and MedHi intake were consistently associated with lower odds of obesity (Table S2).

We further examined the relationships between gut‐microbiota–related dietary patterns and traditional anthropometric measures. Regression analyses indicated that DI‐GM and MedHi intake were significantly associated with lower BMI (Table S3) and smaller WC (Table S4).

Finally, to explore the potential mediating role of serum vitamin D, mediation analyses were performed. Figure S1A illustrates that vitamin D partially mediates the association between DI‐GM and obesity, while Figure S1B shows a similar mediating effect for dietary live microbe intake. Collectively, these sensitivity analyses confirmed the consistency and robustness of the primary associations observed for visceral fat obesity in the main analyses.

## 5 Discussion

Our analysis of 8080 US adults revealed inverse associations between gut microbiota‐targeted dietary patterns and VFO, with vitamin D mediating approximately 17% of these protective effects.

Our results demonstrated a clear inverse association between gut microbiota–targeted dietary patterns and visceral adiposity. These findings are consistent with accumulating evidence that dietary components modulate gut microbiota composition and function, thereby influencing host metabolic health (Boughanem et al. 2023; Zmora et al. 2019; Perler et al. 2023). Specifically, fiber‐rich and plant‐based diets foster microbial diversity and the production of beneficial metabolites such as short‐chain fatty acids (SCFAs), which exert anti‐inflammatory and insulin‐sensitizing effects (Goldsmith and Sartor 2014; Koh et al. 2016). Notably, the inverse associations between DI‐GM/MedHi intake and VFO persisted in individuals with BMI < 30. This observation reinforces the concept of “normal‐weight obesity” introduced earlier, and highlights that gut‐microbiota–targeted diets may confer metabolic protection even before overt obesity develops.

The observed mediation by vitamin D is mechanistically plausible. Vitamin D influences gut barrier integrity, microbial composition, and immune modulation through activation of the vitamin D receptor (VDR) (Vernia et al. 2022). Experimental studies suggest that VDR activation promotes the growth of beneficial commensals and suppresses pro‐inflammatory pathways (Wu et al. 2015; Chen et al. 2015). In the context of obesity, low vitamin D levels are frequently associated with dysbiosis, increased gut permeability, and endotoxemia, all of which may contribute to visceral fat accumulation (Valle et al. 2021; Argano et al. 2023). The vitamin D‐mediated pathway may operate through several mechanisms. First, vitamin D enhances intestinal barrier function by upregulating tight junction proteins, thereby reducing lipopolysaccharide translocation and systemic inflammation (Wu et al. 2015; Zhang et al. 2022; Kong et al. 2008). Second, VDR activation in adipocytes inhibits pre‐adipocyte differentiation and promotes adipocyte apoptosis, particularly in visceral depots (Blumberg et al. 2006; Abbas 2017). Third, vitamin D modulates gut microbiota composition, promoting butyrate‐producing bacteria that enhance insulin sensitivity and reduce visceral fat accumulation through SCFA‐mediated signaling (Luthold et al. 2017; Boughanem et al. 2023).

Moreover, results from multiple sensitivity analyses further strengthened the robustness of our findings. DI‐GM and MedHi intake showed consistent associations with general obesity, BMI as a continuous measure, and WC. In addition, mediation analyses demonstrated that vitamin D partially mediated these associations across different adiposity phenotypes, as shown in Figure S1A,B. Collectively, these supplementary analyses confirm that the observed relationships between gut microbiota–targeted dietary patterns and visceral adiposity are stable across various model specifications and adiposity indicators.

These findings reinforce the importance of dietary strategies that promote gut microbial health—specifically, increased fiber, polyphenols, and live microbes—in combating central obesity. With the gut microbiome increasingly recognized as a therapeutic target (Marchesi et al. 2016), our study adds to a growing body of evidence supporting microbiota‐directed dietary interventions. The partial mediation by vitamin D suggests that combined modulation of microbial and vitamin D pathways may offer synergistic benefits.

Although the observed associations between gut microbiota–targeted dietary patterns and visceral fat area were statistically significant, the magnitude of these associations was relatively modest. This is not unexpected in population‐based observational studies of habitual dietary exposures, where estimated effect sizes represent average differences across a nationally representative sample rather than clinically meaningful changes at the individual level. Nevertheless, the consistency of associations across multiple adiposity‐related outcomes, including VFA, BMI, and WC, supports the internal coherence of the findings. Accordingly, these results should be interpreted as reflecting modest population‐level associations rather than direct evidence of clinically significant effects. From this perspective, the findings highlight the potential relevance of considering gut microbiota–targeted dietary patterns within stratified and phenotype‐oriented approaches to metabolic health. As the field progresses, future longitudinal and interventional studies are warranted to determine whether gut microbiota–based dietary indices can be meaningfully integrated into personalized nutrition strategies and obesity prevention programs.

The strengths of this study include the use of a nationally representative sample, standardized VFO assessment, and adjustment for a broad range of confounders. In addition, the mediation analyses offer supportive evidence regarding the potential intermediary role of vitamin D in the associations between gut microbiota–targeted dietary patterns and VFO.

Nonetheless, several limitations should be noted. First, the cross‐sectional nature of the NHANES data precludes the establishment of temporal sequence and therefore limits causal interpretation of the observed associations. Second, dietary and vitamin D assessments were based on single‐time‐point data, subject to recall and seasonal variability. Third, while the DI‐GM and live microbe intake indices capture key microbiota‐supportive dietary patterns, they do not directly reflect gut microbiota composition, functional activity, or the actual viability of microbes at the point of consumption. Classification of dietary live microbe intake was based on food type and processing characteristics rather than direct measurement of microbial viability; the number and viability of live microbes may therefore vary depending on storage, preparation, and processing conditions. Such exposure misclassification is likely to be non‐differential with respect to visceral fat obesity and would tend to bias associations toward the null. Fourth, we did not directly measure gut microbiota composition, limiting our ability to confirm whether dietary indices truly reflected microbial diversity and function. Fifth, the single 24‐h dietary recall may not capture habitual dietary patterns, potentially introducing measurement error. Sixth, although mediation analyses were performed to explore the potential intermediary role of serum vitamin D, the cross‐sectional design of NHANES precludes establishment of temporal sequence. Therefore, the observed mediation should be interpreted as statistical rather than causal mediation. Reverse causality is also possible, as increased visceral adiposity may contribute to lower circulating vitamin D concentrations through sequestration in adipose tissue or volumetric dilution (Karampela et al. 2021). Longitudinal studies are needed to clarify the temporal and causal relationships among diet, vitamin D status, and visceral fat accumulation.

## 6 Conclusions

In conclusion, this study demonstrates that higher adherence to gut microbiota–targeted dietary patterns, as reflected by DI‐GM and live microbe intake, is inversely associated with VFA accumulation among U.S. adults. Serum vitamin D was identified as a partial statistical mediator of these associations. Together, these findings highlight potential links among diet, vitamin D status, and VFO at the population level, and underscore the need for future longitudinal and interventional studies to clarify temporal relationships and clinical relevance.

## Author Contributions

**Huiling Zheng:** writing – original draft, methodology, validation. **Yan Wang:** funding acquisition, investigation, writing – review and editing, supervision, resources, conceptualization.

## Funding

This work was supported by grants from the Support Project for Clinical Specialty Capacity Building of the First Affiliated Hospital, Sun Yat‐sen University (R7003102) and Guangdong Provincial Medical Science Research Fund Project (C2020050).

## Conflicts of Interest

The authors declare no conflicts of interest.

## Supporting information


**Figure S1:** Mediation analysis models for DI‐GM (A) and live microbe intake (B) with vitamin D as a mediator of obesity (BMI ≥ 30).

**Table S1:** fsn371530‐sup‐0002‐Tables.docx.

## Data Availability

The data that support the findings of this study are openly available in the National Health and Nutrition Examination Survey (NHANES) at https://www.cdc.gov/nchs/nhanes/index.htm.
