# Supplementary figures and images for: The Dietary Index for Gut Microbiota and Live Microbe Intake in Relation to Visceral Fat Obesity: Evidence From NHANES With Vitamin D as a Mediator
Source: Food Sci Nutr. 2026 Feb 12;14(2):e71530. doi: 10.1002/fsn3.71530 (PMC12900904; doi:10.1002/fsn3.71530)

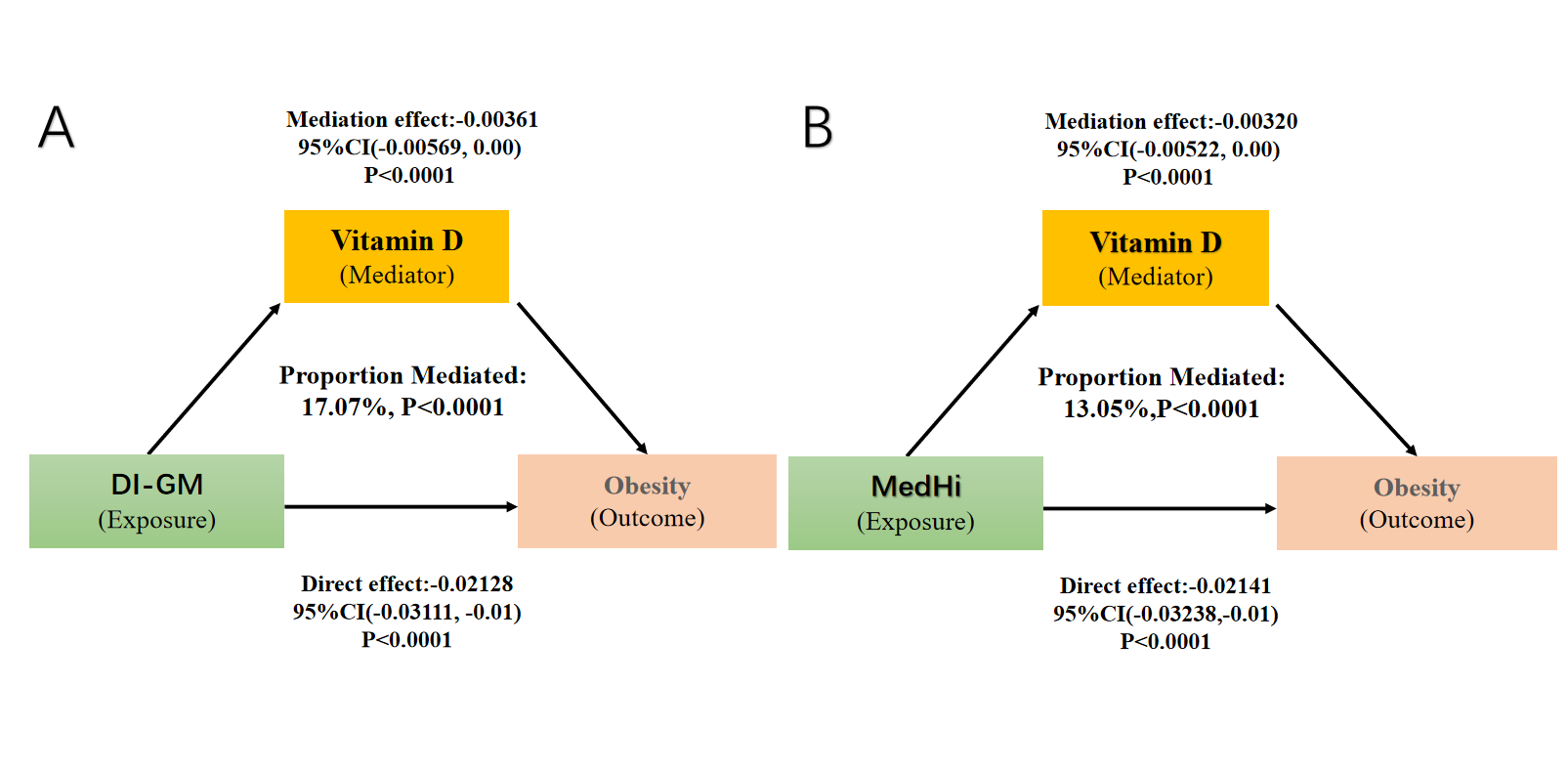

Supplement: Supplementary file 1 — Figure S1: Mediation analysis models for DI‐GM (A) and live microbe intake (B) with vitamin D as a mediator of obesity (BMI ≥ 30). [file FSN3-14-e71530-s001.png]
