# Supplementary material for: The Dietary Index for Gut Microbiota and Live Microbe Intake in Relation to Visceral Fat Obesity: Evidence From NHANES With Vitamin D as a Mediator
Source: Food Sci Nutr. 2026 Feb 12;14(2):e71530. doi: 10.1002/fsn3.71530 (PMC12900904; doi:10.1002/fsn3.71530)
Supplement: Supplementary file 2 — Table S1: fsn371530‐sup‐0002‐Tables.docx. [file FSN3-14-e71530-s002.docx]

| Supplementary Table 1. Associations between DI-GM, MedHi intake and VFO among participants with BMI < 30 | | | | | | | | |
| --- | --- | --- | --- | --- | --- | --- | --- | --- |
|  | **Model 1 OR (95%CI)** | **P value** | **Model 2 OR (95%CI)** | **P value** | **Model 3 OR (95%CI)** | **P value** | **Model 4 OR (95%CI)** | **P value** |
| **DI_GM groups** |  |  |  |  |  |  |  |  |
| Q1 | 1.00 (Reference) |  | 1.00 (Reference) |  | 1.00 (Reference) |  | 1.00 (Reference) |  |
| Q2 | 1.10(0.84,1.43) | 0.47 | 0.99(0.72,1.36) | 0.93 | 0.96(0.66,1.39) | 0.83 | 0.99(0.69,1.42) | 0.96 |
| Q3 | 0.96(0.76,1.21) | 0.7 | 0.71(0.54,0.93) | **0.01** | 0.70(0.51,0.96) | **0.03** | 0.72(0.52,0.99) | **0.04** |
| Q4 | 0.76(0.55,1.04) | 0.09 | 0.39(0.26,0.58) | **<0.0001** | 0.41(0.26,0.63) | **<0.001** | 0.45(0.29,0.70) | **<0.001** |
| **p for trend** |  | 0.28 |  | **<0.001** |  | **0.002** |  | **0.008** |
| **MedHi groups** |  |  |  |  |  |  |  |  |
| G1 | 1.00 (Reference) |  | 1.00 (Reference) |  | 1.00 (Reference) |  | 1.00 (Reference) |  |
| G2 | 0.95(0.77,1.17) | 0.63 | 0.77(0.54,1.12) | 0.17 | 0.74(0.50,1.08) | 0.11 | 0.72(0.49,1.06) | 0.09 |
| G3 | 0.77(0.67,0.88) | **<0.001** | 0.64(0.49,0.82) | **<0.001** | 0.63(0.46,0.86) | **0.01** | 0.66(0.48,0.92) | **0.02** |
| **p for trend** |  | 0.404 |  | **<0.001** |  | **0.006** |  | **0.018** |
| Model 1 was crude model. Model 2 was adjusted for age and gender. Model 3 was adjusted for Model 2, and race, PIR, educational level,alcohol use,smoking status,eGFR, ALT，TC，Protein intake, Energy intake,Vitamin D intake,Hypertension,DM and PA. Model 4 was adjusted for Model 3, and serum vitamin D. PIR: poverty income ratio; eGFR: estimated glomerular filtration rate; ALT:alanine aminotransferase,TC:total cholesterol,PA: physical activity; DM**：**Diabetes Mellitus；DI_GM:Dietary Index for Gut Microbiota;OR, odds ratio; CI, confidence interval. | | | | | | | | |

| Supplementary Table 2. Associations between DI-GM, MedHi intake and general obesity (BMI ≥ 30) | | | | | | | | |
| --- | --- | --- | --- | --- | --- | --- | --- | --- |
| Variable | **Model 1 OR (95%CI)** | **P value** | **Model 2 OR (95%CI)** | **P value** | **Model 3 OR (95%CI)** | **P value** | **Model 4 OR (95%CI)** | **P value** |
| **DI_GM groups** |  |  |  |  |  |  |  |  |
| Q1 | 1.00 (Reference) |  | 1.00 (Reference) |  | 1.00 (Reference) |  | 1.00 (Reference) |  |
| Q2 | 1.03(0.88,1.21) | 0.71 | 1.02(0.87,1.19) | 0.81 | 1.02(0.84,1.24) | 0.84 | 1.04(0.86,1.27) | 0.68 |
| Q3 | 0.79(0.68,0.92) | **0.002** | 0.76(0.65,0.89) | **<0.001** | 0.84(0.71,1.00) | **0.05** | 0.88(0.74,1.04) | 0.13 |
| Q4 | 0.50(0.38,0.65) | **<0.0001** | 0.47(0.36,0.61) | **<0.0001** | 0.58(0.44,0.76) | **<0.001** | 0.63(0.48,0.83) | **0.002** |
| **p for trend** |  | **<0.001** |  | **<0.001** |  | **0.014** |  | **0.044** |
| **MedHi groups** |  |  |  |  |  |  |  |  |
| G1 | 1.00 (Reference) |  | 1.00 (Reference) |  | 1.00 (Reference) |  | 1.00 (Reference) |  |
| G2 | 1.12(0.92,1.36) | 0.27 | 1.12(0.92,1.37) | 0.26 | 1.13(0.90,1.43) | 0.28 | 1.15(0.91,1.45) | 0.24 |
| G3 | 0.66(0.56,0.77) | **<0.0001** | 0.63(0.54,0.74) | **<0.0001** | 0.68(0.57,0.81) | **<0.0001** | 0.71(0.60,0.85) | **<0.001** |
| **p for trend** |  | **<0.0001** |  | **<0.0001** |  | **<0.0001** |  | **<0.001** |
| Models 1-4 were adjusted as described in Supplementary Table 1. | | | | | | | | |

| Supplementary Table 3. Regression analyses showing the associations of DI-GM and MedHi (per 100 g) with BMI | | | | | | | | |
| --- | --- | --- | --- | --- | --- | --- | --- | --- |
| Variables | Model 1 | *P* | Model 2 | *P* | Model 3 | *P* | Model 4 | *P* |
| DI-GM | -0.4(-0.52,-0.29) | **<0.0001** | -0.46(-0.58,-0.34) | **<0.0001** | -0.29(-0.41,-0.17) | **<0.0001** | -0.22(-0.35,-0.10) | **<0.001** |
| MedHi(per 100g) | -0.34(-0.45,-0.23) | **<0.0001** | -0.37(-0.48,-0.26) | **<0.0001** | -0.26(-0.38,-0.14) | **<0.001** | -0.21(-0.32,-0.10) | **<0.001** |

Models 1-4 were adjusted as described in Supplementary Table 1.

| Supplementary Table 4. Regression analyses showing the associations of DI-GM and MedHi (per 100 g) with WC | | | | | | | | |
| --- | --- | --- | --- | --- | --- | --- | --- | --- |
| Variables | Model 1 | *P* | Model 2 | *P* | Model 3 | *P* | Model 4 | *P* |
| DI-GM | -0.99(-1.26,-0.71) | **<0.0001** | -1.1(-1.38,-0.82) | **<0.0001** | -0.77(-1.06,-0.48) | **<0.0001** | -0.62(-0.93,-0.32) | **<0.001** |
| MedHi(per 100g) | -0.88(-1.10,-0.66) | **<0.0001** | -0.94(-1.15,-0.72) | **<0.0001** | -0.68(-0.95,-0.40) | **<0.0001** | -0.55(-0.80,-0.31) | **<0.0001** |
| Models 1-4 were adjusted as described in Supplementary Table 1. | | | | | | | | |
